# Synchronous online learning during movement control order in higher education institutions: a systematic review

**DOI:** 10.12688/f1000research.73342.2

**Published:** 2021-11-19

**Authors:** Yee Wan Lee, Magiswary Dorasamy, Abdul Aziz Bin Ahmad, Manimekalai Jambulingam, Peik Foong Yeap, Sharbani Harun

**Affiliations:** 1 Faculty of Management, Multimedia University, Cyberjaya, Selangor, 63100, Malaysia; 2 Taylor's University, Subang Jaya, Selangor, 47500, Malaysia; 3 University of Newcastle, Callaghan, NSW, 2308, Australia; 4 Technology Park Malaysia, Kuala Lumpur, Wilayah Persekutuan, 57000, Malaysia

**Keywords:** Interest, Engagement, Synchronous Online Learning, Higher Education Institution, Movement Control Order, Pandemic, Covid-19, Malaysia

## Abstract

**Background:** Higher education institutions (HEI) are not spared from the coronavirus disease 2019 (COVID-19) pandemic. The closure of campuses because of the movement control order (MCO) to mitigate the spread of the COVID-19 has forced HEIs to adopt online learning, especially synchronous online learning (SOL). Although teaching and learning can be continued via SOL, retaining students’ interest and sustaining their engagement have not been sufficiently explored. This study presents a systematic review of the research pertaining to SOL associated with students’ interest and engagement in HEIs during the MCO environment.

**Methods**: Five major online databases, i.e., EBSCOhost, Science Direct, Emerald, Scopus and Springer were searched to collect relevant papers published between 1st January 2010 to 15th June 2021 including conference proceedings, peer-reviewed papers and dissertations. Papers written in the English language, based in full-fledged universities, and with these five keywords: (i) synchronous online learning, (ii) engagement, (iii) interest, (iv) MCO/Covid-19 and (v) HEI, were included. Papers focussing on synchronous and asynchronous online learning in schools and colleges were excluded. Each paper was reviewed by two reviewers in order to confirm the eligibility based on the inclusion and exclusion criteria.

**Results**: We found 31 papers of which six papers were related to SOL, engagement and interest in HEIs in the MCO environment. Our review presents three major findings: (i) limited research has been conducted on SOL associated with students’ engagement and interest, (ii) studies related to the context of HEIs in the MCO environment are limited, and (iii) the understanding of the new phenomena through qualitative research is insufficient. We highlight the SOL alignment with students’ engagement, interest, style preference, learner interaction effectiveness, behavior and academic performance.

**Conclusions:** We believe that the findings of this study are timely and require attention from the research community.

## Introduction

The Malaysian online learning movement started in the 1990s with the objective of providing learners access to quality education and lifelong learning opportunity. ^1^ Despite the growing online learning trend, physical learning remains the mainstream learning mode for full-time undergraduate students because most of the Malaysian universities’ infrastructures, facilities and program structures are built for physical teaching and learning. ^2^ However, the eruption of the coronavirus disease 2019 (COVID-19) pandemic in 2019 changed this norm. The enforcement of movement control order (MCO) in Malaysia has pushed universities towards online learning. Therefore, synchronous online learning (SOL) has been adopted as a temporary solution in the time of the pandemic to ensure the continuity of academic activities. Even though literature abounds regarding online learning for adult learners in the normal environment, synchronous online learning (SOL) for undergraduate students during the COVID-19 pandemic is a new phenomenon that warrants the attention of research community. Therefore, a systematic literature reviews will help the researchers to identify the research gaps in this new environment.

### Synchronous online learning (SOL)

SOL is a form of online learning where teaching and learning occur simultaneously and at the same place. ^3^ In SOL, students and instructors can login remotely from any location in the world and concurrently participate in the learning process. ^4^^,^^5^ The advancement of online learning technologies, such as audio, video, and text, has allowed instant feedback and real-time interaction between students, instructors and fellow students. ^6^^-^^11^ These features of SOL that resemble physical learning are well accepted by students. ^12^^,^^13^ Despite the benefits of live session, immediacy and real-time guidance and feedback, SOL has its limitations. ^6^^,^^14^^,^^15^ For instance, technical difficulties, availability of electronic devices, internet connection, interface and bandwidth and students’ interest and engagement are issues related to SOL in higher education institutions (HEIs) in the MCO environment. ^16^

### Engagement

Engagement is referred to as the interaction between the time, effort and relevant resources invested to optimize student’s experience, learning outcomes and performance. ^17^ Engagement is also related to student’s attitudes towards the learning process and psychological involvement in the learning activities to attain positive learning outcomes, such as satisfaction, achievement and performance. ^18^^,^^19^ Behavioral, cognitive and emotional engagement are the three main engagement components. ^20^ Behavioral engagement requires students to comply with the behavioral norms, where students do not demonstrate disruptive or negative behavior. Students with positive behavioral engagement will attend classes and participate enthusiastically in the learning process. Next, students with emotional engagement demonstrate interest and enjoyment in the learning process. Lastly, students with cognitive engagement will go the extra mile in the learning process to perform beyond expectation.

### Interest

Interest is the underlying psychological factor of being engaged or engrossed in an activity and a guiding factor in energising learning and academic performance. ^21^^,^^22^ Next, continuing interest requires students to endure and reengage in the learning activities over time. ^23^ Therefore, educational activities that meet individual students’ needs can catch students’ attention, such as by varying the novelty, complexity and incongruity of visual stimuli. ^21^^,^^22^^,^^24^ Nevertheless, the heterogeneity of individual interest and the large class size have made these tasks challenging. ^25^ Hence, creating situational interest in the learning process is the first step in developing students’ individual interest ^26^^,^^27^ because students with a positive individual interest are highly engaged and attentive to achieve good academic performance as individual interest is a psychological behavior of positive affect and persistency in the learning process. ^28^^,^^29^ When students’ individual interest matches the specific contextual affordances, students will be focused and enjoy learning. A study suggests that interest will develop into a self-sustained and well-developed interest with the passage of time. ^21^

Given this backdrop, the three research questions for this study are as follows:

1. Do research gaps in SOL pertaining to students’ engagement and interest in HEIs in the MCO environment exist?
2. What are the limitations in the current research within SOL in HEIs in the MCO environment?
3. What is the conceptual framework for SOL pertaining to students’ engagement and interest in HEIs in the MCO environment?

The objectives of this proposal are as follows:

1. To identify research gaps in SOL pertaining to students’ engagement and interest in HEIs in the MCO environment.
2. To understand the limitations of the current research within SOL in HEIs in the MCO environment.
3. To develop a conceptual framework for SOL pertaining to students’ engagement and interest in HEIs in the MCO environment.

## Methods

### Institutional review board statement

This study was approved by the Research Ethical Committee (REC) of Multimedia University (EA2742021).

### Study design

This paper was designed to present a literature review, a research gap analysis and insights into SOL pertaining to undergraduate students’ engagement and interest in HEIs in the MCO environment. The five stages of literature review proposed by ^30^ were used in this process:

Stage 1: Planning the review

Stage 2: Identifying and evaluating studies

Stage 3: Extracting and synthesising data

Stage 4: Reporting descriptive findings

Stage 5: Utilising the findings to inform research and practice

This review is reported in line with the Preferred Reporting Items for Systematic Reviews and Meta-Analyses (PRISMA) reporting guidelines. ^62^

### Stage 1. Planning the review

The main purpose of this review is to identify research gaps in terms of theories, factors, methods and processes pertaining to SOL and the engagement and interest elements in HEIs in the MCO environment.

### Stage 2. Identifying and evaluating studies

The main focus of this study is SOL. Therefore, papers on asynchronous learning were excluded in the selection and evaluation process. Nevertheless, the identification and evaluation were hampered because not all the papers used the term synchronous and asynchronous explicitly.

**Selection process**

**Figure 1.**
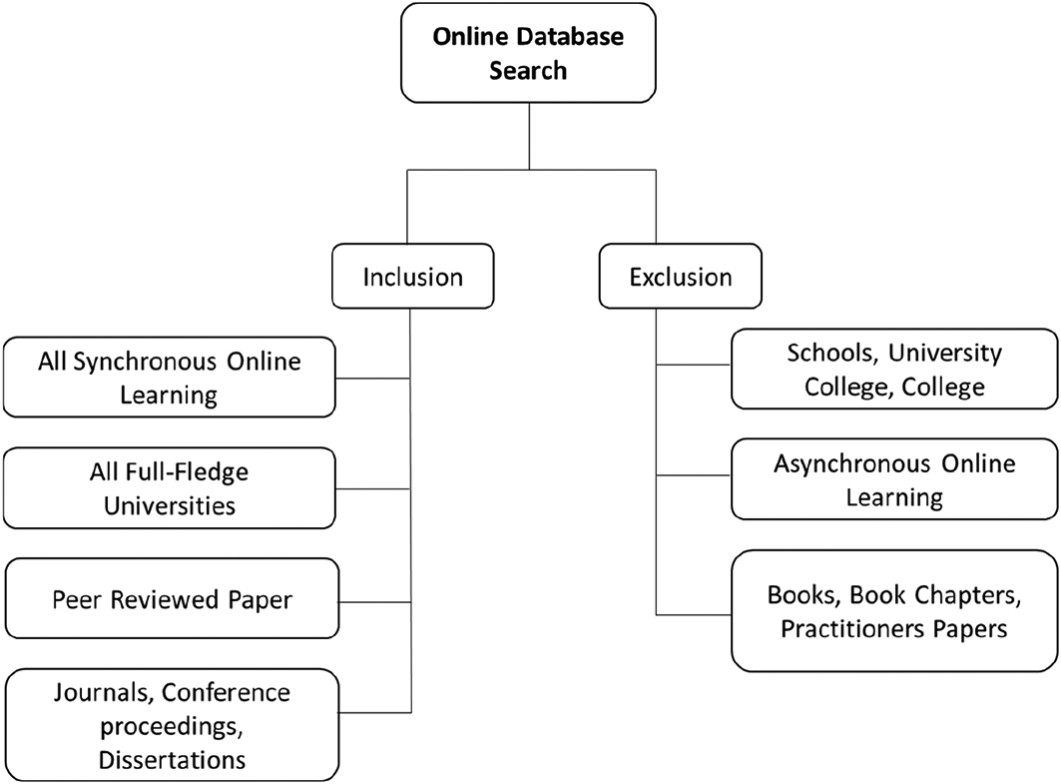
Paper selection process flow.

Five major online databases, namely EBSCOhost, Science Direct, Emerald, Scopus and Springer were searched to collect relevant papers published between 1 ^st^ January 2010 to 15 ^th^ June 2021 including conference proceedings, peer-reviewed papers and dissertations. Papers written in the English language and based in full-fledged universities with these five keywords: (i) synchronous online learning, (ii) engagement, (iii) interest, (iv) MCO/COVID-19 and (v) HEI, were included in the selection process. Papers that focused on synchronous and asynchronous online learning in schools and colleges were excluded. The limitation of this selection process strategy is that other terminologies used for online learning such as hybrid-classroom, flipped-classroom, distance learning, E-learning, Massive Open Online Course (MOOC) and etc. were excluded. Similarly, terms such as lockdown, circuit-breaker and etc. were not included in the search process. Figure 1 presents the paper selection process. Next, each paper was reviewed by two reviewers collectively in order to confirm the eligibility based on the inclusion and exclusion criteria.

**Search strategy**

The search strategy was to sift through papers that discuss SOL pertaining to students’ engagement and interest in the time of MCO using the combination of keywords ( Table 1). However, the search strategy that based on the five identified keywords in the selection process may lead to publication bias risk. Therefore, future researchers may expand the selection of keywords in order to reduce the risk of missing some relevant articles.

**Table 1.** Search strategy.

Synchronous online learning (SOL)	Synchronous online learning (SOL)	Synchronous online learning (SOL)	Synchronous online learning (SOL)	Synchronous online learning (SOL)	Synchronous online learning (SOL)	Synchronous online learning (SOL)	Date search
	Engagement	Interest	Engagement	Engagement	Engagement	Engagement	1 June 2021 to 15 June 2021
			Interest	Interest	Interest	Interest	1 June 2021 to 15 June 2021
				Coronavirus disease 2019 (COVID-19)	Coronavirus disease 2019 (COVID-19)	Coronavirus disease 2019 (COVID-19)	1 June 2021 to 15 June 2021
					Movement control order (MCO)	Movement control order (MCO)	1 June 2021 to 15 June 2021
						Higher education institutions (HEIs)	1 June 2021 to 15 June 2021

### Stage 3. Extracting and synthesising data

In this extraction process, only conceptual and empirical papers associated with SOL, engagement and interest in HEIs under MCO were selected for synthesis. Further, data on theories, methods, factors and limitations of the selected papers were reviewed and evaluated. Table 2 presents the focus areas in extracting and synthesising data from the selected papers.

**Table 2.** Paper search focus areas.

Focus areas	Theory	Method	Factor	Limitation
Definition	What theory/theories were used in this research?	What research method/methods were used to collect research data in this research?	What factor/factors were used to explain the research?	What were the limitation/limitations of this research?

**Figure 2.**
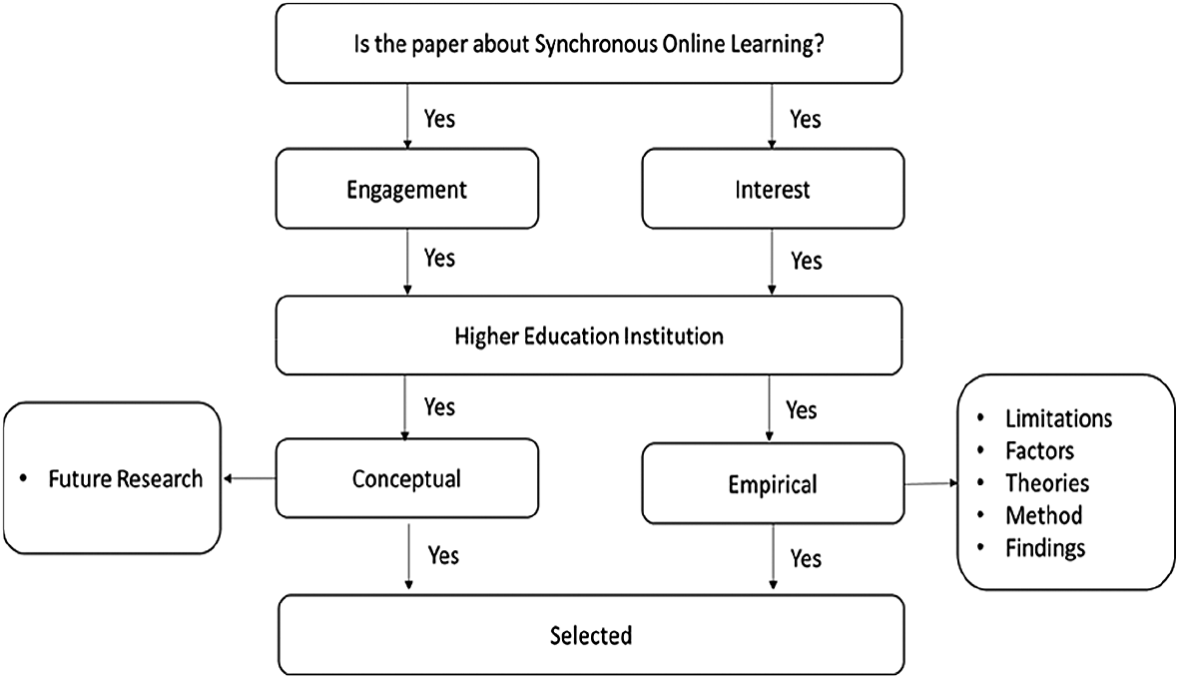
Data extraction and synthesis process.

Figure 2 presents the papers extraction process.

The Transfield stages 4 and 5 are presented in the following sections.

## Results

### Reporting descriptive findings

Table 3 presents the online databases search result. A total of 21,431 papers are listed as “synchronous online learning”. The number dropped to 4,970 (23%) after adding the word “engagement” and dropped further to 2,020 (9.4%) after adding the word “interest”.

**Table 3.** Summary of keyword search result.

Keywords combinations		Synchronous online learning	Synchronous online learning	Synchronous online learning	Synchronous online learning	Synchronous online learning	Synchronous online learning	Synchronous online learning	Date search
			Engagement	Interest	Engagement	Engagement	Engagement	Engagement	6th June 2021
					Interest	Interest	Interest	Interest	6th June 2021
						Covid-19	Covid 19	Covid-19	6th June 2021
							MCO	MCO	6th June 2021
								HEI	6th June 2021
**No**	**Online database**								
1	EBSCOhost	263	26	1	0	0	0	0	6th June 2021
2	Science direct	11659	1808	3391	1078	67	17	7	6th June 2021
3	Scopus	393	22	0	0	0	0	0	6th June 2021
4	Springer	8116	2251	360	159	14	8	7	6th June 2021
5	Emerald	1000	863	0	783	72	6	6	6th June 2021
	Grand total	21431	4970	3752	2020	153	31	20	

We examined 31 papers. However, as shown in Table 4, only six out of the 31 papers are associated with SOL in HEI in the MCO environment. Further, only two out of these six papers are related to engagement, and no paper is related to the interest element.

**Table 4.** Data extraction and synthesis process.

No	Combination of keywords	Frequency
1	Synchronous online learning (SOL) + Higher education institutions (HEIs)	6
2	Synchronous online learning (SOL) + Higher education institutions (HEIs) + Movement control order (MCO)	6
3	Synchronous online learning (SOL) + Higher education institutions (HEIs) + Movement control order (MCO) + Engagement	2
4	Synchronous online learning (SOL) + Higher education institutions (HEIs) + Movement control order (MCO) + Interest	0
5	Synchronous online learning (SOL) + Higher education institutions (HEIs) + Movement control order (MCO) + Engagement + Interest + Qualitative methods	0
6	Qualitative methods	7

Table 5 summarizes the theories, methods and factors used in these 31 papers. Only 8 papers (26%) used qualitative methods, 8 papers (26%) are associated with SOL, 5 papers (16%) are related to engagement, no paper (0%) is related to interest, 11 papers (35%) are related to HEI and 19 papers (61%) are related to MCO. However, only 2 papers (6%) were associated to SOL, engagement, HEI and MCO. Therefore, the research in the area of student’s engagement and interest, particularly that associated with SOL in HEI in the MCO environment.

**Table 5.** Summary of 31 selected papers. SOL = synchronous online learning; MCO = movement control order; HEI = higher education institution.

No	Citation	Author (Year)	Theory	Method	Factor	Limitation	Has the paper discussed about these keywords?				
							SOL	Engagement	Interest	MCO	HEIs
1	31	Aguilera-Hermida (2020)		Quantitative	Attitude, Affect, Motivation; Perceived behavioral control Cognitive engagement	No standardized questions were asked. Only students from public universities were included. Study excluded students who did not have access to the Internet.	x	x		x	x
2	33	Srivastava *et al*. (2021)		Quantitative	Anxiety level		x			x	x
3	37	Darling-Aduana (2021)		Mixed method	Authentic work (Course videos, assignments, practice problems, and assessments)	Components of authentic work, such as communication and interaction with peers, that were not facilitated by the online course system evaluated					x
4	38	Bogdan *et al*. (2021)		Qualitative	Protective and precautionary behaviors, social connections, and self-efficacy						
5	39	Li *et al*. (2020)			5G, AI, AR					x	
6	40	Corbin (2020)	The Stairway to Lifetime Fitness, Health, and Wellness		Conceptual physical education (CPE)					x	
7	42	Tyerman, Luctkar-Flude, & Baker (2021)		Qualitative						x	
8	43	Constantin *et al* (2021)		Qualitative	Participatory Design (PD)			x		x	
9	44	Philippe *et al*. (2020)		Qualitative	Serious games, Simulations, Collaborative VR	lack of robust evaluation framework					
10	45	Thomas *et al*. (2021)	Behavioral lifestyle intervention	Experiment	Virtual reality, Interactive video feedback, Tailored intervention						
11	46	Shankar *et al*. (2021)			Technology adoption (customers, suppliers, employees, retailers)	Investigate the impact of technology on not just retail outcomes but also on the whole retail ecosystem				x	
12	47	Keswani, Brooks & Khoury (2020)			Supervision, Virtual mentoring, Virtual classroom, Didactic curriculum						
13	48	Khodadad-Saryazdi (2021)		Qualitative	Adoption, Routinization, Implementation						
14	49	Tessitore *et al*. (2021)		Systematic literature review		Deliberate exclusion of articles related to support from other sources					
15	41	Abdelgaffar (2021)		Qualitative							x
16	50	Applin & Flick (2021)			Public behaviors						
17	51	Fernandez-Álvarez *et al*. (2020)	Encyclopaedia								
18	52	Ironsi (2021)		Quantitative		Obtaining ethical consent from the participants was difficult as well and so the sample size was small.	x				
19	32	Ali, Narayan & Sharma (2020)	Engagement	Qualitative			x	x		x	x
20	53	Badiozaman, Leong & Wong (2020)		Quantitative	Online teaching and learning, mastering Google Classroom, cloud-based productivity tools, netiquette, cybersecurity	Small sample size - Digital Educator Series training, the accuracy of the description may be unique to this particular group of individuals.	x			x	
21	35	Tan (2020)		Quantitative	Motivation, Community of inquiry, cognitive presence and teaching presence and Learning performance.		x			x	x
22	36	Simoes *et al*. (2021)		Quantitative	Availability of resources, Virtual learning process, Performance of students		x			x	x
23	54	López *et al*. (2020)								x	
24	55	Kundu & Bej (2021)		Quantitative	Pedagogies, challenges faced		x	x		x	
25	56	Romero-Hall (2021)			Digital divide, Internet filtering policies, lack of research, Education reform, M-learning technology, Social media ethics						x
26	57	Caligiuri *et al*. (2020)								x	
27	58	Pacheco (2020)								x	
28	59	Moura, Nascimento & Ferreira (2021)		Conceptual						x	
29	34	Ismailov & Ono (2021)	Motivation, Self-Determination theory, Expectancy-value theory	Quantitative	Motivation	Study involved groups of mainly first-year Japanese students, and thus, the sample may have been rendered homogeneous	x	x		x	x
30	60	Chang & Kuo (2021)	Cultural Historical activity theory		Interactions and interactivity (LMS)					x	x
31	61	Leal Filho *et al*. (2021)		Quantitative						x	x

Table 6 presents the plotting of 31 papers to engagement, interest, synchronous online learning (SOL), movement control order (MCO) and higher education institutions (HEIs).

**Table 6.** Plotting of papers regarding engagement, interest, SOL (synchronous online learning), HEI (higher education institutions), and MCO (movement control order).

No	Author (Year)	Has the paper discussed about these keywords?				
		Synchronous online learning (SOL)	Engagement	Interest	Movement control order (MCO)	Higher education institutions (HEIs)
1	Aguilera-Hermida (2020)	x	x		x	x
2	Srivastava *et al*. (2021)	x			x	x
3	Darling-Aduana (2021)					x
4	Bogdan *et al*. (2021)					
5	Li *et al*. (2020)				x	
6	Corbin (2020)				x	
7	Tyerman, Luctkar-Flude, & Baker (2021)				x	
8	Constantin (2021)		x		x	
9	Philippe (2020)					
10	Thomas (2021)					
11	Shankar (2021)				x	
12	Keswani, Brooks, & Khoury (2020)					
13	Khodadad-Saryazdi (2021)					
14	Tessitore (2020)					
15	Abdelgaffar (2021)					x
16	Applin, & Flick (2021)					
17	Fernandez-Álvarez, (2020)					
18	Ironsi (2021)	x				
19	Ali, Narayan & Sharma (2020)	x	x		x	x
20	Badiozaman, Leong & Wong (2020)	x			x	
21	Tan (2020)	x			x	x
22	Simoes *et al*. (2021)	x			x	x
23	López *et al*. (2020)				x	
24	Kundu & Bej (2021)	x	x		x	
25	Romero-Hall (2021)					x
26	Caligiuri *et al*. (2020)				x	
27	Pacheco (2020)				x	
28	Moura, Nascimento & Ferreira (2021)				x	
29	Ismailov & Ono (2021)	x	x		x	x
30	Chang & Kuo (2021)				x	x
31	Leal Filho *et al*. (2021)				x	x

**Figure 3.**
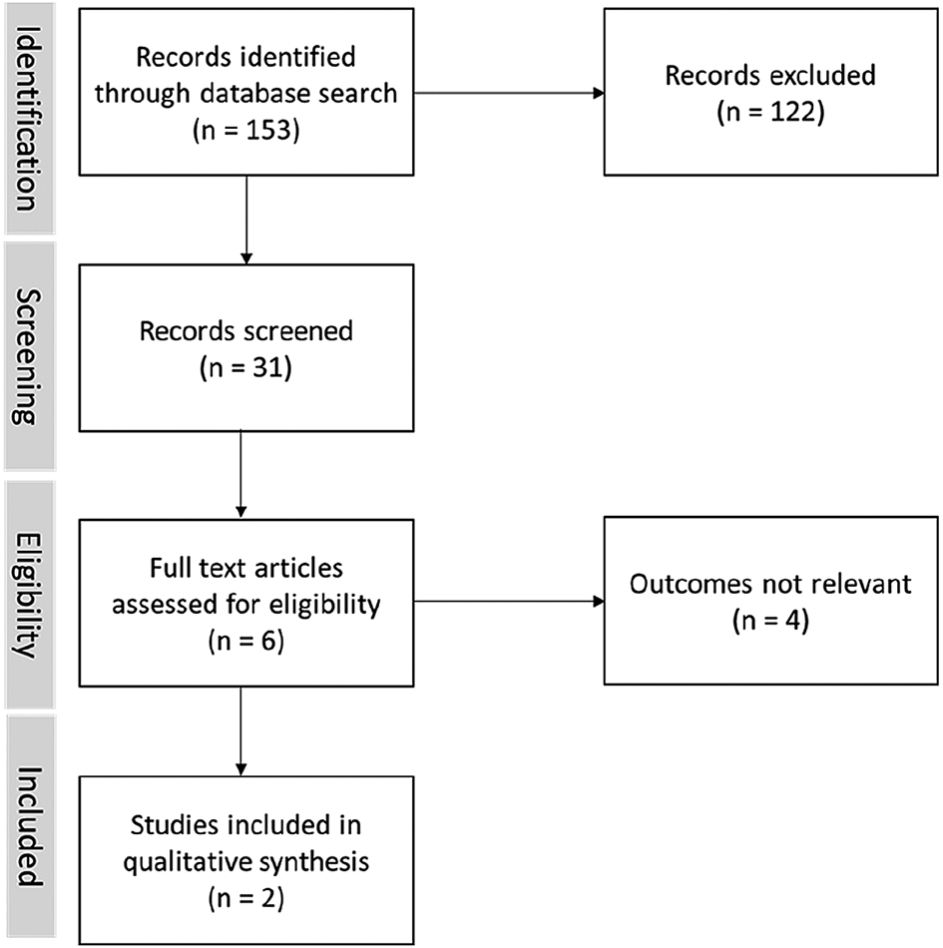
Paper search records.

Table 5 and Table 6 show that, student’s engagement and interest associated with SOL in HEIs in the MCO environment is insufficiently researched. The online databases search results in Figure 3 also shows that only two papers are relevant to our research.

## Discussion

Table 7 presents the summary of the six papers related to synchronous online learning (SOL) in higher education institutions (HEIs) in the movement control order (MCO) environment.

**Table 7.** Summary of six papers related to SOL (synchronous online learning) + HEI (higher education institutions) + MCO (movement control order). COVID-19 = coronavirus disease 2019.

No	Citation	Author (Year)	Paper description
1	31	Aguilera-Hermida (2020)	This paper explores college students’ perception of their adoption, use and acceptance of emergency online learning, particularly their attitude, affect, motivation and perceived behavioral control and cognitive engagement using quantitative data collection methods.
2	33	Srivastava *et al*. (2021)	This paper aims to evaluate medical students’ anxiety levels and its correlation with academic factors during emergency remote learning using a questionnaire survey. Research findings show that about one-fourth of medical students have anxiety issue during emergency remote learning.
3	32	Ali, Narayan & Sharma (2020)	This paper aims to provide insights on students’ engagement in the learning of accounting subject during the COVID-19 disruption and the pivot to online learning based on reflections of academic staff members teaching the accounting subject at two large New Zealand universities. The findings of this paper suggests that there are some successes and challenges in engaging students in online learning of accounting subject.
4	34	Ismailov & Ono (2021)	This paper aims to examine factors that are influencing Japanese college freshmen’ motivation when completing graded online assignments as part of the English reading courses during COVID-19 pandemic using qualitative method.
5	35	Tan (2020)	This paper focuses on university students by analyzing students’ motivation, the community of inquiry and learning performance using quantitative analysis and paired sample t-test. Research findings indicated that due to the lack of learning infrastructures to support online learning and social support, students’ motivation and learning performance are affected.
6	36	Simoes *et al*. (2021)	This paper aims to analyze biological engineering students’ adaptation to virtual learning environment during COVID-19 pandemic. Research findings show that there is an overall improvement in students’ performance despite changes made to pedagogical like course design, teaching method and evaluation.

Based on the above summary, research in the areas of undergraduate students’ engagement and interest in synchronous online learning during movement control order warrant further investigation. In-depth research into these areas will help the research community to offer practical solutions to synchronous online learning during MCO for undergraduate students who are used to physical face-to-face learning to increase learning effectiveness during the time of a crisis, such as a Coronavirus pandemic crisis. Next, this research will also bridge the theoretical gap where new or enhanced conceptual framework can be introduced.

### Research gaps

Three major research gaps were identified:

***Research gap 1: SOL in HEI during MCO context***

Only six papers ^31^^–^^36^ are related to HEI in the MCO environment. Therefore, this new phenomenon warrants further investigation by the research community.

***Research gap 2: Engagement and interest elements***

Only two papers ^31^^,^^32^ related to engagement are associated with SOL in the MCO environment. The study on SOL associated with engagement and interest is clearly insufficient. Moreover, Paper 1 ^31^ focused only on students’ cognitive engagement, such as attitude, affect, and motivation, whereas Paper 2 ^32^ focused on accounting lecturers’ reflection regarding students’ engagement during synchronous and asynchronous online classes. Engagement can be categorized into cognitive, behavioral and emotional engagement. ^20^ A holistic view on students’ engagement should include these three dimensions. Next, students’ interest in learning via SOL will affect the quality of engagement. Nevertheless, interest as a factor associated with SOL in HEI in the MCO environment has not been researched intensively. Therefore, the inclusion of interest warrants the attention of the research community.

***Research gap 3: Method***

In total, seven qualitative research methods were identified from the 31 papers. Only one paper used case study method. SOL in HEIs in the MCO environment is a new phenomenon that may require a more in-depth investigation method, such as a case study method to gain better insights.

### Limitations in current studies within synchronous online learning (SOL) in higher education institutions (HEIs) in the movement control order (MCO) environment

Table 8 presents the following limitations of the six papers pertaining to SOL associated with engagement and interest in HEI in the MCO environment.

**Table 8.** Limitations of the papers.

No	Citation	Author (Year)	Limitation
1	31	Patricia Aguilera-Hermida (2020)	No standardized questions were asked. Only students from public universities were included. Study excluded students who did not have access to the Internet.
2	33	Srivastava *et al*. (2021)	
3	37	Darling-Aduana (2021)	Components of authentic work, such as communication and interaction with peers, that were not facilitated by the online course system evaluated
4	38	Bogdan *et al*. (2021)	
5	39	Li *et al*. (2020)	
6	40	Corbin (2020)	
7	42	Tyerman, Luctkar-Flude, & Baker (2021)	
8	43	Constantin *et al* (2021)	
9	44	Philippe *et al*. (2020)	Lack of robust evaluation framework
10	45	Thomas *et al*. (2021)	
11	46	Shankar *et al*. (2021)	Investigate the impact of technology on not just retail outcomes but also on the whole retail ecosystem
12	47	Keswani, Brooks & Khoury (2020)	
13	48	Khodadad-Saryazdi (2021)	
14	49	Tessitore *et al*. (2021)	Deliberate exclusion of articles related to support from other sources
15	41	Abdelgaffar (2021)	
16	50	Applin & Flick (2021)	
17	51	Fernandez-Álvarez *et al*. (2020)	
18	52	Ironsi (2021)	Obtaining ethical consent from the participants was difficult and so the sample size was small.
19	32	Ali, Narayan & Sharma (2020)	
20	53	Badiozaman, Leong & Wong (2020)	Small sample size - Digital Educator Series training, the accuracy of the description may be unique to this particular group of individuals.
21	35	Tan (2020)	
22	36	Simoes *et al*. (2021)	
23	54	López *et al*. (2020)	
24	55	Kundu & Bej (2021)	
25	56	Romero-Hall (2021)	
26	57	Caligiuri *et al*. (2020)	
27	58	Pacheco (2020)	
28	59	Moura, Nascimento & Ferreira (2021)	
29	34	Ismailov & Ono (2021)	Study involved groups of mainly first-year Japanese students, and thus, the sample may have been rendered homogeneous.
30	60	Chang & Kuo (2021)	
31	61	Leal Filho *et al*. (2021)	

Four limitations were identified from the 31 selected papers:

i. These papers mainly focused on the cognitive dimension of engagement. Therefore, the inclusion of behavioral and emotional dimensions may be important for a holistic understanding of students’ engagement. Next, engagement is a personal factor. Therefore, investigating students directly to gauge their engagement level is important.
ii. Few papers related to the interest element pertaining to SOL in the MCO environment exist. Interest is closely associated with engagement in the learning process. Therefore, interest must be included in the study of SOL in HEI in the MCO environment.
iii. An in-depth understanding of how students are engaged, and their interest sustained through case study research will help complement the findings derived from quantitative studies pertaining to SOL associated with engagement and interest in HEI in the MCO environment.
iv. Based on the identified 31 papers, only 6 papers were related to the targeted research areas. The rest of the 25 papers were irrelevant to the research areas. For instance, student’s anxiety level during MCO, the used of 5G technology in online education, proficiency usage of various online teaching platforms and devices and etc.

**Figure 4.**
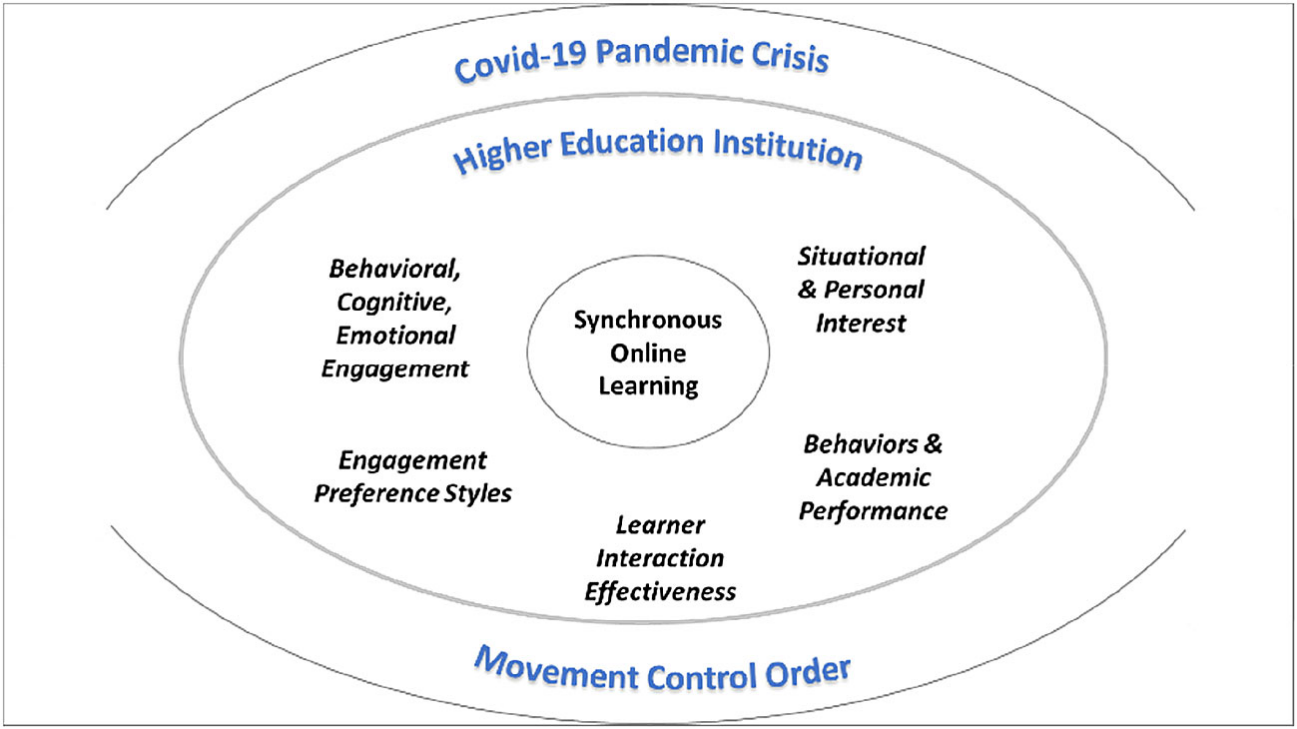
Conceptual framework. COVID-19 = coronavirus disease 2019.

Therefore, Figure 4 is the conceptual framework for students’ engagement and interest pertaining to SOL in HEI in the MCO environment.

## Future recommendations

Given the findings and discussions for this systematic literature review, Table 9 presents recommendations for future research in association with SOL in HEI under MCO.

**Table 9.** Future research recommendations.

No	Future recommendations
1	Synchronous online learning (SOL) in higher education institutions (HEIs) in the movement control order (MCO) environment in terms of students’ engagement preference styles.
2	Synchronous online learning (SOL) in higher education institutions (HEIs) in the movement control order (MCO) environment in terms of effectiveness of interaction between student and student, between student and instructor and student with content.
3	Synchronous online learning (SOL) in higher education institutions (HEIs) in the movement control order (MCO) environment in terms of students’ behaviors and academic performance.

## Conclusions

This systematic literature review highlighted three research gaps associated with SOL in HEI in the MCO environment and four limitations were identified from the 31 selected research papers. A conceptual framework is proposed for future research related to synchronous online learning, engagement and interest in the movement control environment. Practical solutions can be identified to improve the effectiveness of teaching and learning in this new phenomenon.

## Data availability

### Underlying data

All data underlying the results are available as part of the article and no additional source data are required.

### Reporting guidelines

Figshare: PRISMA flow diagram and checklist for ‘Synchronous online learning during movement control order in higher education institutions: a systematic review. https://doi.org/10.6084/m9.figshare.16752031.v1. ^62^

Data are available under the terms of the Creative Commons Attribution 4.0 International license (CC-BY 4.0).
