# Supplementary material for: Synchronous online learning during movement control order in higher education institutions: a systematic review
Source: F1000Res. 2021 Nov 19;10:1056. Originally published 2021 Oct 18. [Version 2] doi: 10.12688/f1000research.73342.2 (PMC8666982; doi:10.12688/f1000research.73342.2)
Supplement: Peer review history

# Peer review: Reviewer response for version 2

**DOI:** 10.5256/f1000research.79516.r100708

**Published:** 2021-12-07

**Authors:** Jessica Sze Yin Ho

**Affiliations:** 1 Heriot-Watt University, Putrajaya, Malaysia

I have looked through the revision and I’m happy with the work that the authors have put in. I would like to recommend that the paper should be accepted.

Are the rationale for, and objectives of, the Systematic Review clearly stated?

Yes

Is the statistical analysis and its interpretation appropriate?

Partly

Are sufficient details of the methods and analysis provided to allow replication by others?

Partly

Are the conclusions drawn adequately supported by the results presented in the review?

No

Reviewer Expertise:

Digital Marketing, higher education, sustainability

I confirm that I have read this submission and believe that I have an appropriate level of expertise to confirm that it is of an acceptable scientific standard.

# Peer review: Reviewer response for version 1

**DOI:** 10.5256/f1000research.76986.r97281

**Published:** 2021-11-09

**Authors:** Jessica Sze Yin Ho

**Affiliations:** 1 Heriot-Watt University, Putrajaya, Malaysia

This study is an interesting study in view of the lockdown situation. The paper is also written in a very straight-forward manner that makes it easy to follow.

 Some additional comments on the paper:

1. SOL is also sometimes used in different terminologies for example hybrid-classroom. Has this been incorporated as a search term? If not, including this in the limitation of this research will be helpful for future research. 
2. Similarly, MCO is a local term. It is good to see that COViD-19 has been used as a keyword. To appeal to a larger audience, other terms such as lockdown or circuit-breaker may have been studied globally. Adding more papers incorporating these search terms can help to attract a larger international audience. Justifying this will be essential for this study.
3. Generally, the findings of this paper is rather limited. The ultimate findings were based on 6 papers. Are there any other literature gaps that can be identified in this systematic literature review based in the 31 papers identified? 
4. More critical discussion on the findings is expected - implication to theory and practice. 
5. Conclusion - needs enhancement. 

Are the rationale for, and objectives of, the Systematic Review clearly stated?

Yes

Is the statistical analysis and its interpretation appropriate?

Partly

Are sufficient details of the methods and analysis provided to allow replication by others?

Partly

Are the conclusions drawn adequately supported by the results presented in the review?

No

Reviewer Expertise:

Digital Marketing, higher education, sustainability

I confirm that I have read this submission and believe that I have an appropriate level of expertise to confirm that it is of an acceptable scientific standard, however I have significant reservations, as outlined above.

# Peer review: Reviewer response for version 1

**DOI:** 10.5256/f1000research.76986.r97295

**Published:** 2021-11-02

**Authors:** Manjula Nagarajan

**Affiliations:** 1 Department of Management Studies, Thiagarajar School of Managemnet, Madurai, Tamil Nadu, India

The authors have done a very good work. During Pandemic ( the MCO Environment) the learning process was the most hit and children were really suffering across globe. This systematic review would help authors to get into an in-depth research of SOL. Hence the area and the topic of the research is well connected with the need of the hour.

 In this paper the analysis is done well and it is suggested that the authors can add on a country-wise analysis of the research papers. This could give us an idea as to which continent had contributed to this area of research more and would have paved way for increasing the scope of the research.

Are the rationale for, and objectives of, the Systematic Review clearly stated?

Yes

Is the statistical analysis and its interpretation appropriate?

Not applicable

Are sufficient details of the methods and analysis provided to allow replication by others?

Yes

Are the conclusions drawn adequately supported by the results presented in the review?

Yes

Reviewer Expertise:

Synchronous Online learning (SOL), Impact of the learning process during the pandemic, impact of SOL on the Case teaching method a pedagogy in Management education

I confirm that I have read this submission and believe that I have an appropriate level of expertise to confirm that it is of an acceptable scientific standard.
